# Effects of acotiamide on esophageal motor function and gastroesophageal reflux in healthy volunteers

**DOI:** 10.1186/s12876-015-0346-7

**Published:** 2015-09-11

**Authors:** Norihisa Ishimura, Mami Mori, Hironobu Mikami, Shino Shimura, Goichi Uno, Masahito Aimi, Naoki Oshima, Shunji Ishihara, Yoshikazu Kinoshita

**Affiliations:** Department of Gastroenterology and Hepatology, Shimane University School of Medicine, 89-1, Enya-cho, Izumo, Shimane 693-8501 Japan

## Abstract

**Background:**

The prevalence of gastroesophageal reflux disease (GERD) has been increasing worldwide, with proton pump inhibitor (PPI) administration the current mainstay therapy for affected individuals. However, PPI efficacy is insufficient especially for non-erosive reflux disease. Although it has been reported that prokinetic drugs improve GERD, their effects on esophageal function remain to be clearly investigated. In the present study, we evaluated the direct effects of acotiamide, a novel prokinetic agent for the treatment of functional dyspepsia, on esophageal motor function and gastroesophageal reflux.

**Methods:**

Ten adult healthy volunteers (average age 24 years, range 20–36 years; 7 males, 3 females) were enrolled. Esophageal body peristaltic contractions and lower esophageal sphincter (LES) pressure with and without acotiamide administration were recorded using high resolution manometry using a cross-over protocol. Total and acidic reflux levels for 24 h and during the postprandial period were also recorded using a multichannel intraluminal impedance and pH monitoring system. Data were analyzed blind by one observer.

**Results:**

Acotiamide at a standard dose of 300 mg/day did not significantly stimulate esophageal motor function. Although the frequency of swallows with weak contraction tended to decrease with acotiamide administration, the difference as compared to no administration was not statistically significant. In addition, the drug neither decreased total or postprandial gastroesophageal acid/non-acid reflux events nor accelerated esophageal clearance time.

**Conclusions:**

Acotiamide, a novel gastrointestinal motility modulator, at a standard dose did not significantly affect esophageal motor functions or gastroesophageal reflux in healthy adults. Additional investigations with GERD patients are necessary to elucidate its clinical significance.

**Trial registration:**

This study was registered on 1st August 2013 with the University Hospital Medical Information Network (UMIN) clinical trials registry, as number: UMIN000011260.

## Background

Gastroesophageal reflux disease (GERD) is the most common gastrointestinal disorder worldwide, with a prevalence of 10–30 % in Western countries and 5–10 % in Asia [1]. Despite geographical variations, the prevalence of GERD has continued to increase [2]. In Japan as well, GERD has become more prevalent in recent decades [3, 4], mainly due to the westernization of eating habits, increased number of obese individuals and gastric acid secretion, and decreased rate of *Helicobacter pylori* infection [5–8]. GERD significantly reduces health-related quality of life (QOL), resulting in a marked economic burden on health care systems [9]. Moreover, severe and long-lasting GERD is an important risk factor for esophageal adenocarcinoma [10, 11] the incidence of which has risen rapidly over the past 3 decades in Western countries [12]. Therefore, management of GERD by drug administration is pivotal for these related issues.

Medical anti-reflux treatment, most notably with proton pump inhibitors (PPIs), is the current mainstay therapy for GERD [13]. Presently, PPIs are the most effective class of drugs for relieving GERD-related symptoms, as well as healing and maintaining remission of erosive esophagitis, and improving health-related QOL. Despite its efficacy for treating GERD and GERD-related complications, use of a PPI alone remains insufficient for many GERD patients, as overall 30 % of GERD patients, 10–15 % of erosive esophagitis patients, and 40–50 % of non-erosive reflux disease (NERD) patients do not experience symptom alleviation with conventional PPI therapy [14, 15].

Prokinetic drugs are presumed to improve GERD by increasing lower esophageal sphincter (LES) basal pressure, enhancing esophageal peristalsis, accelerating esophageal acid clearance, and facilitating gastric emptying. These include 5-hydroxytryptamine (5-HT) receptor agonists, GABA-B receptor agonists, dopamine receptor antagonists, and others [16, 17]. Although many studies have shown that addition of a prokinetic to PPI therapy can improve GERD symptoms, some controversy remains in literature [18, 19], while the effects of prokinetics on esophageal function are also controversial [18, 20].

Acotiamide is a novel upper gastrointestinal motility modulator recently approved in Japan for treatment of functional dyspepsia (FD) [21, 22]. This drug enhances acetylcholine release from enteric neurons through muscarinic receptor antagonism and acetylcholinesterase (AchE) inhibition, thereby enhancing gastric emptying and gastric accommodation [23]. In addition, acotiamide was reported to stimulate not only gastric antral motility, but also duodenal and colonic motility during the postprandial state in conscious dogs [24]. However, the direct effects of acotiamide on esophageal motor function have not been well elucidated. In the present study, we assessed the effects of acotiamide on esophageal motor functions and gastroesophageal reflux (GER) in healthy adults to determine its therapeutic potential for GERD.

## Methods

### Enrolled subjects

Ten adult healthy volunteers (7 males, 3 females; mean age 24 years, range 20–36 years) were recruited for this study. None of the subjects had upper gastrointestinal symptoms, history of upper gastrointestinal surgery, or were taking medications known to influence esophageal motor function. Written informed consent was obtained from each before starting the study, which was carried out in accordance with the Declaration of Helsinki. The present study was approved by the ethics committee of Shimane University School of Medicine. This study was registered with the University Hospital Medical Information Network (UMIN) clinical trials registry, number UMIN 000011260.

### Study protocol

**Fig. 1:**
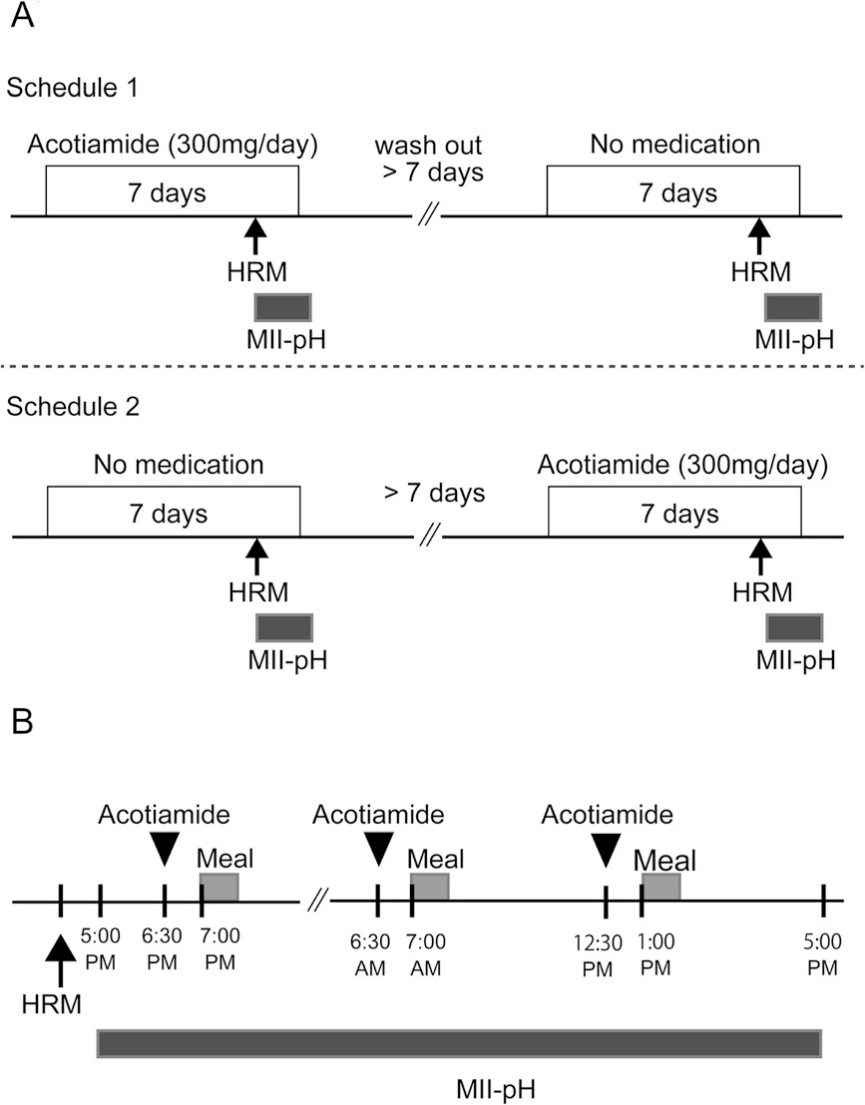
**a** Study protocol. Esophageal motor function and gastroesophageal reflux were evaluated after a 7-day administration of acotiamide (300 mg/day) or no medication using a cross-over protocol. On the day before the last day of administration, determinations of esophageal motor function were performed by high resolution manometry (HRM). After finishing the HRM, 24-hour esophageal multichannel intraluminal impedance and pH testing (MII-pH) were conducted. The 2 trials (with and without acotiamide) were conducted at least 1 week apart and in random order. **b** Schedule on the day of HRM and MII-pH

Esophageal motor function and GER were evaluated after a 7-day administration of acotiamide or no medication using a cross-over protocol (Fig. 1). Acotiamide (Acofide®, Zeria Pharmaceutical Co., Ltd. and Astellas Pharma Inc., Tokyo, Japan) at 100 mg was administrated with 100 mL of water 3 times/day at 30 min before each meal for 7 days, which is commonly used for adult patients with FD in Japan. As a control, the same volunteers were given 100 mL of water before each meal for 7 days. On the day before the last day of administration, determinations of esophageal motor function and GER were performed. The 2 trials (with and without acotiamide) were conducted at least 1 week apart and in random order.

### Esophageal high-resolution manometry (HRM)

High-resolution manometric tests were conducted using a ManoScan 360™ system (Sierra Scientific Instruments, Inc., Los Angeles, CA) [25]. HRM was performed at 4 h after administration of acotiamide, which was appropriate for evaluation of its effects [26]. The HRM catheter used with this system is 4.2 mm in diameter and has 36 intraluminal pressure transducers at 1-cm intervals, which are used to simultaneously and continuously measure peristaltic pressure in the area from the upper esophageal sphincter (UES) to the LES. The HRM catheter was inserted in a transnasal manner using 2 % lidocaine jelly (Xylocaine jelly; AstraZeneka Co., Osaka, Japan), then LES pressure was measured in a supine position during a 5-minute rest period. Next, esophageal body peristaltic function in the supine position was examined by swallowing 5 mL of room temperature water, which was repeated at 30 s intervals until 10 recordings of complete esophageal peristalsis were obtained. After finishing the tests in the supine position, they were repeated in a sitting position. Peristaltic contractions in the esophageal body were divided into 3 different segments (1, 2, and 3 in order from oral to anal) separated by 2 troughs [27–29]. LES pressure and peak intraesophageal contraction pressure in the 3 segments of the esophageal body were analyzed using ManoView™ analysis software (Sierra Scientific Instruments). Contractile front velocity (CFV) (cm/s), defined as the slope of the tangent approximating the 30-mmHg isobaric contour between the proximal pressure trough and contractile deceleration point, was also determined [30].

To evaluate the effect of acotiamide on esophageal peristalsis, the integrity of contraction associated with each swallow in the supine position was assessed. According to the updated Chicago classification of esophageal motility disorders [31], contraction pattern was divided into 3 categories; intact, premature, and fragmented contraction. Fragmented contraction was defined as normal integrity with large break (>5 cm in length) in the 20 mmHg isobaric contour. We also assessed the number of normal integrity with small break (2–5 cm in length) in the 20 mmHg isobaric contour [30]. Finally, minor disorders of peristalsis was divided into 2 groups; ineffective esophageal motility (≥50 % ineffective swallows) and fragmented peristalsis (≥50 % fragmented contraction), based on the frequency of swallows with ineffective or fragmented contraction [31].

### Assessment of gastroesophageal reflux

After finishing the esophageal HRM examination, 24-hour esophageal multichannel intraluminal impedance and pH testing (MII-pH) were conducted using an MII-pH monitoring system (Sandhill Scientific Inc., Highlands Ranch, CO). We utilized a 2.1-mm diameter combined MII-pH catheter (Sandhill Scientific Inc.) equipped with 6 impedance and 2 (esophageal and gastric) antimony pH sensors with an external reference. Prior to the procedure, the pH sensors were calibrated using solution buffered at pH 4.0 and 7.0, according to the manufacturer’s instructions. The probe was then inserted in a transnasal manner through the esophagus into the stomach and the esophageal pH sensor was positioned at 5 cm above the upper limit of the LES. The design of the probe allowed measurement of impedance data at 3, 5, 7, 9, 15, and 17 cm above the LES. The catheter was connected to a data logger (Sleuth System; Sandhill Scientific Inc.) that stored data from the 8 channels (6 impedance, 2 pH) at a frequency of 50 Hz.

After insertion of the MII-pH catheter, a standardized high calorie meal was given to the subjects for dinner at 30 min after acotiamide administration. They were instructed to ingest the high calorie meal consisting of a large plate of curry and rice, along with cheese soup, which totalled 1067 kcal, with 27.2 g of protein, 160.2 g of carbohydrates, and 34.6 g of fat, within 30 min. The next day, standardized breakfast and lunch meals were also served at 30 min after acotiamide administration (Fig. 1b). After 24-hour esophageal MII-pH monitoring, the MII-pH data were downloaded and analyzed using dedicated software (Bio View Analysis; Sandhill Scientific Inc.) after a manual analysis of each MII-pH tracing. Bolus clearance time was defined as lapsed time that the bolus was present at each impedance level during a specific reflux episode or time interval between bolus entry and clearance. Acid exposure time was calculated as the percentage of time during which pH was below 4. A total of acid exposure time of ≥4 % was considered to be pathological [32]. The numbers of total and postprandial GER events were counted for 24 and 6 h (sum of 2 h after each meal), respectively. Reflux episodes were further classified as acidic or non-acidic, as previously described [33]. As a control, the same procedures were conducted without administration of acotiamide, with 100 mL of water given instead at 30 min before each meal.

### Statistical analysis

Data were analyzed blind by one observer (H.M.) and expressed as median (interquartile range [IQR]). Statistical analyses were performed using a chi-square test and Wilcoxon signed rank test. All calculations were conducted using the SPSS statistical package 20.0 (IBM SPSS Japan Inc., Tokyo, Japan), with differences at *p* <0.05 considered to be statistically significant.

## Results

### Effects of acotiamide on esophageal motor function

**Fig. 2:**
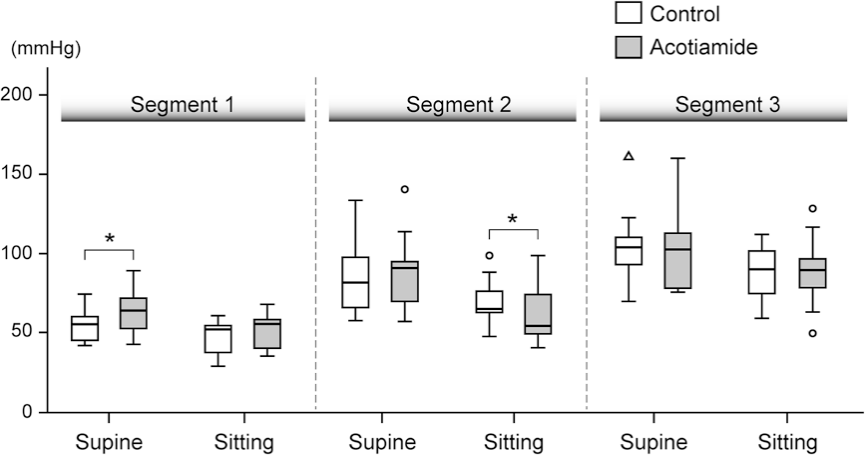
Peak peristaltic contraction pressure in 3 different segments of the esophageal body with and without acotiamide administration. Contraction pressures were weaker in the sitting position than in the supine position. Administration of acotiamide induced slight changes in segment 1 and 2, while there was no significant change in segment 3, which is most related to esophageal peristaltic contractions. *p < 0.05, significantly different

All 10 subjects completed the study protocol without any adverse events. Resting LES pressure with and without acotiamide in the supine and sitting positions were measured. Consistent with a previous report, LES pressure determined in the supine position was significantly greater than in the sitting position [34]. However, there were no statistically significant difference for the LES pressure values determined with and without administration of acotiamide in both positions (Table 1). The peak peristaltic pressures in the 3 segments of the esophageal body in the supine position were significantly higher than those in the sitting position, and also significantly increased from segment 1 to 3 (Table 1, Fig. 2). Furthermore, the peak peristaltic pressure in segment 1 in the supine position with acotiamide was significantly higher than without medication (*p* <0.05). On the other hand, the peak peristaltic pressure in segment 2 in the sitting position with acotiamide was significantly lower than without medication (*p* <0.05). Values for the other peak contraction pressures, including segment 3, the most important in relation to esophageal peristalsis, did not show significant differences among them. Likewise, there was no significant difference for CFV observed with acotiamide administration (Table 1). Collectively, acotiamide did not show significant augmentation of esophageal contractions.

**Table 1:** Parameters during esophageal body contractions with and without administration of acotiamide

	Supine position		*p* value	Sitting position		*p* value
	Control	Acotiamide		Control	Acotiamide	
Segment 1 (mmHg)	55.4 [46.0–59.7]	64.1 [55.0–70.8]	0.02	52.8 [40.8–54.7]	56.1 [42.8–58.0]	0.18
Segment 2 (mmHg)	81.9 [68.5–96.1]	90.5 [72.8–93.7]	0.33	65.3 [62.8–75.8]	54.3 [49.7–71.9]	0.04
Segment 3 (mmHg)	103.9 [93.7–109.4]	106.7 [84.4–124.5]	0.58	89.9 [77.0–99.7]	88.8 [75.4–93.5]	0.96
LES pressure (mmHg)	19.8 [18.0–25.1]	21.9 [18.4–24.1]	0.88	15.0 [11.4–21.0]	14.3 [8.5–20.9]	0.58
CFV (cm/s)	5.7 [4.6–6.1]	5.5 [4.5–6.5]	0.96	5.0 [4.2–6.5]	4.8 [4.4–5.4]	0.58

*LES* Lower esophageal sphincter, *CFV* Contractile front velocity. Values are expressed as median. Number of brackets show interquartile range of each pattern

### Effects of acotiamide on esophageal peristalsis

All the subjects (*n* = 10) showed normal esophageal motility assessed by the updated Chicago Classification [31], and the pattern did not change with acotiamide administration. In addition, no subjects had any esophageal symptoms during the study period.

Next, we assessed the frequency of swallows with abnormal contraction with or without administration of acotiamide. Of 10 subjects, 4 had no change with or without administration of acotiamide. Five subjects improved the frequency of swallows with abnormal contraction, mostly weak contraction with small break to normal with administration of acotiamide, while one had increased frequency of abnormal contraction. Collectively, the frequency of swallows with weak contraction, including large and small break, tended to decrease with acotiamide administration [13.4 % (IQR 0–28.6) vs 0 % (IQR 0–12.5)], though the difference as compared to no administration was not statistically significant (*p* <0.14).

### Effects of acotiamide on gastroesophageal reflux

**Fig. 3:**
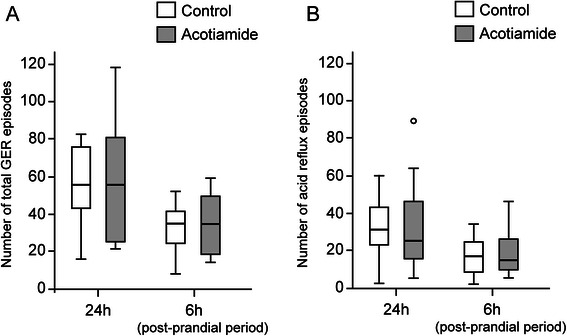
Gastroesophageal reflux (GER) events with and without acotiamide administration. The number of total GER events was not different between with and without acotiamide administration after both 24 h and 6 h (postprandial period) (**a**). Likewise, the number of acid refluxes did not differ between the groups for both periods (**b**)

The values for percentage time at intraesophageal pH <4.0 for 24 and 6 h (sum of 2-hour postprandial periods) were determined. Of 10 subjects, excessive acid reflux, which was defined as time at pH <4.0 exceeded 4 % of the total recording time, was present in one subjects, who remained excessive reflux with acotiamide administration (7.8 % vs 5.8 %). Collectively, there was no significant difference in percent of esophageal acid exposure time between with and without acotiamide administration, as well as in both the 24-hour examination and postprandial period (Table 2). In addition, the number of total refluxes observed was not different between with and without acotiamide in the 24- and 6-hour (postprandial) periods (Table 2, Fig. 3). Likewise, the number of acid refluxes did not differ between the groups for both periods. Furthermore, the bolus clearance time of gastroesophageal refluxant measured in the lower esophagus 5 cm above the LES did not significantly show any difference between the groups (Table 2).

**Table 2:** Assessment of gastroesophageal reflux with and without administration of acotiamide

	24 h			Post prandial (6 h)		
	Control	Acotiamide	*p* value	Control	Acotiamide	*p* value
pH <4 (%)	1.1 [0.5–2.0]	0.6 [0.3–2.4]	0.47	2.0 [0.3–3.3]	2.0 [0.8–2.6]	0.99
Number of reflux episodes						
Total reflux	56.0 [44.0–76.5]	56.0 [25.0–81.5]	0.96	34.0 [23.5–40.5]	34.0 [18.0–48.5]	0.58
Acid reflux	31.0 [23.0–43.5]	25.0 [15.5–46.0]	0.76	17.0 [8.5–24.5]	15.0 [10.5–26.0]	0.45
Bolus clearance time (s)	14.0 [12.0–15.0]	14.0 [13.0–18.5]	0.11	14.0 [12.5–17.5]	16.0 [14.5–19.0]	0.17

Values are expressed as median. Number of brackets show interquartile range of each pattern

## Discussion

In the present study, we determined whether a standard dose of acotiamide directly stimulates esophageal motor function to prevent the occurrence of GER in healthy adults. Our findings are the first to show that acotiamide does not enhance esophageal motor functions or prevent GER events.

Impaired esophageal motor functions, such as decreased LES pressure and weak esophageal body peristalsis, are primary causes of GERD. It has been proposed that prokinetic agents improve GERD by increasing LES basal pressure, improving esophageal peristalsis, accelerating esophageal acid clearance, and facilitating gastric emptying. Indeed, prokinetic therapy with mosapride or metoclopramide in addition to PPI administration is an option often considered for patients with incomplete response to PPIs [13, 35]. However, the effects of prokinetic agents on esophageal motor function and GERD remain controversial. Previously, we evaluated the effects of several prokinetic agents, including mosapride, itopride, and herbal medicine (TJ-43), on esophageal motor functions [20, 36, 37]. None of those at a standard dose strikingly stimulated esophageal motor activity or decreased postprandial gastroesophageal acid reflux in healthy subjects. In contrast, we and other groups have reported that high-dose mosapride (30–40 mg/day) significantly augmented esophageal motor activities in healthy subjects [38–41] which suggested that higher doses directly augment esophageal body peristaltic contractions and facilitate the esophageal clearance mechanism.

Acotiamide is a novel prokinetic drug based on a mechanism of action that differs from other gastroprokinetic agents, which have little affinity for serotonin 5-HT_2_, 5-HT_3_, and 5-HT_4_ receptors, and weak affinity for dopamine D_2_ receptors. Acotiamide exerts its gastroprokinetic activity via presynaptic M1 and M2 muscarinic receptor inhibition, resulting in enhanced acetylcholine (Ach) release, and via inhibition of AchE activity in the stomach [42–45]. Through these actions, the drug enhances the availability of Ach released in a synaptic manner, thereby enhancing reflex-controlled motility. Two phase 2 studies of acotiamide have been conducted in Japan with FD patients, which established 100 mg t.i.d. (compared to 50 and 300 mg) as the optimal dose and also identified postprandial fullness, early satiety, and abdominal bloating, which were mainly found in meal-induced postprandial distress syndrome (PDS), as the most responsive symptoms [46]. Furthermore, acotiamide is the first approved therapeutic agent for FD diagnosed by Rome III criteria throughout the world, with initial approval occurring in Japan [21, 22], while it is currently under evaluation for treatment of FD in a phase 3 program being conducted in Europe. Although most related clinical studies have focused on improvement of PDS, a study conducted in Europe showed that heartburn severity in FD patients was significantly decreased with administration of 100 mg of acotiamide as compared with a placebo [47]. However, the effect of acotiamide on the improvement of GERD symptoms in FD patients remains obscure whether direct or indirect effect on esophageal motor function.

A strength of this study is that a detailed investigation of the direct effects of acotiamide on esophageal motor function and GER was conducted by both HRM and MII-pH. Impaired esophageal motor functions, especially, weak esophageal peristalsis with large peristaltic break has been reported to be closely associated with delayed acid clearance and mucosal erosion or chronic cough [48, 49]. Therefore, it would be attractive if acotiamide directly improves esophageal contraction pattern and reduces the length of peristaltic breaks. In the present study, none of the healthy subjects showed major or minor disorders of peristalsis. Although the frequency of swallows with weak contraction with small break tended to decrease with acotiamide administration in enrolled subjects, the difference as compared to no administration was not statistically significant. Recently, the significance of detecting small peristaltic break (2–5 cm in length in 20 mmHg isobaric contour) was questioned, and eliminated as a criterion of abnormal contraction pattern in the updated criteria [31]. Moreover, the parameters for assessment of GER assessed by MII-pH did not significantly change with administration of acotiamide in these subjects. Our results suggested that acotiamide did not directly enhance esophageal contraction in healthy subjects. Interestingly, it was recently reported that prucalopride, another 5-HT_4_ receptor agonist, reduced esophageal acid exposure and accelerated gastric emptying with no effects on the number of reflux episodes or esophageal contractility [50]. Together, these findings suggest that acceleration of gastric emptying reduces the availability of gastric contents during a reflux episode, thereby reducing reflux symptoms without directly affecting esophageal motor functions. In clinical settings, there is considerable overlap of symptoms among patients with FD and GERD [4], suggesting that these two disorders share common pathophysiological mechanisms, such as abnormal gastric motility. Therefore, larger studies of FD patients with GERD symptoms are needed to evaluate whether acotiamide indirectly improve GERD symptoms by acceleration of gastric emptying on that population.

Our study has some limitations. The number of volunteers was limited, and not a placebo controlled randomized trial, though a crossover design was utilized. We administrated acotiamide to healthy young volunteers and not GERD patients, who have frequent GER events. Alternatively, we gave a standardized high calorie diet to the subjects during MII-pH monitoring to induce possible postprandial GER with adjusted dietary fat or protein intake among subjects. In addition, we did not evaluate its efficacy toward GERD symptoms and our findings do not completely eliminate the possibility of using acotiamide as a treatment option for cases with PPI-resistant GERD. Several recent studies have shown that acotiamide does not enhance gastric emptying in healthy volunteers but enhance in FD patients, consistent with animal experiments [51–53], suggesting that the effect of acotiamide may be different between patients with impaired gastrointestinal function and healthy adults. Therefore, a future study with a greater number of subjects including patients with GERD, whose esophageal motor function is impaired, is necessary to confirm the direct or indirect effects of acotiamide on esophageal motor function.

## Conclusions

In conclusion, acotiamide at 300 mg/day did not directly affect esophageal motor functions or GER events. Additional studies are needed to clarify its effects in patients with GERD.

## Abbreviations

Ach: Acetylcholine
AchE: Acetylcholinesterase
CFV: Contractile front velocity
FD: Functional dyspepsia
GER: Gastroesophageal reflux
GERD: Gastroesophageal reflux disease
HRM: High-resolution manometry
5-HT: 5-hydroxytryptamine
IQR: Interquartile range
LES: Lower esophageal sphincter
MII-pH: Multichannel intraluminal impedance and pH testing
NERD: Non-erosive reflux disease
PDS: Postprandial distress syndrome
PPI: Proton pump inhibitor
QOL: Quality of life
UES: Upper esophageal sphincter
